# Viruses infecting common bean (*Phaseolus vulgaris* L.) in Tanzania: A review on molecular characterization, detection and disease management options

**DOI:** 10.5897/AJAR2017.12236

**Published:** 2017-05-04

**Authors:** Beatrice Mwaipopo, Susan Nchimbi-Msolla, Paul Njau, Fred Tairo, Magdalena William, Papias Binagwa, Elisiana Kweka, Michael Kilango, Deusdedith Mbanzibwa

**Affiliations:** 1 Mikocheni Agricultural Research Institute, P. O. Box 6226, Dar es Salaam, Tanzania; 2 Department of Crop Science and Horticulture, Sokoine University of Agriculture, P. O. Box 3005, Morogoro, Tanzania; 3 Agricultural Research Institute -Maruku, P. O. Box 127, Bukoba, Tanzania; 4 Agricultural Research Institute -Selian, P. O. Box 6024, Arusha, Tanzania; 5 Agricultural Research Institute -Uyole, P. O. Box 400, Mbeya, Tanzania

**Keywords:** *Bean common mosaic virus* (BCMV), *Bean common mosaic necrosis virus* (BCMNV), common bean viruses, Tanzania, virus molecular detection

## Abstract

Common bean (*Phaseolus vulgaris* L.) is a major legume crop, serving as a main source of dietary protein and calories and generating income for many Tanzanians. It is produced in nearly all agro-ecological zones of Tanzania. However, the average yields are low (<1000 kg/ha), which is attributed to many factors including virus diseases. The most important viruses of common bean in Tanzania are *Bean common mosaic virus* (BCMV) and *Bean common mosaic necrosis virus* (BCMNV) but other viruses have also been reported. There has never been a review of common bean virus diseases in the country, and the lack of collated information makes their management difficult. Therefore, this review focuses on (1) occurrence of different viruses of common bean in Tanzania, (2) molecular characterization of these viruses, (3) detection tools for common bean viruses in Tanzania and (4) available options for managing virus diseases in the country. Literature and nucleotide sequence database searches revealed that common bean diseases are inadequately studied and that their causal viruses have not been adequately characterized at the molecular level in Tanzania. Increased awareness on common bean virus diseases in Tanzania is expected to result into informed development of strategies for management of the same and thus increased production, which in turn has implication on nutrition and income.

## INTRODUCTION

Common bean (*Phaseolus vulgaris* L.) is a diploid (2n = 2x = 22) self-pollinating species that can also out-cross, albeit at very low rates (Ferreira et al., 2000; Gepts, 2001). It originates from Mesoamerica (Bitocchi et al., 2012). It is documented that common bean was introduced in coastal areas of East Africa, especially Tanzania, in the 16^th^ century by the Portuguese and that further spread in inland areas occurred through the Arab slave traders (Wortmann et al., 2004). Common bean is an essential source of proteins and nutrients to over 500 million people in Africa, Latin America and the Caribbean (Singh, 2005; Cortés et al., 2013). It plays a key role in reducing malnutrition as well as generating income for otherwise low-income households in the developing world.

In Tanzania, beans are commonly cultivated as intercrops with other crops such as banana and maize. They are grown in mid- to high-altitude areas of the country, which experience more reliable rainfall and cooler temperatures (Hillocks et al., 2006). Specifically, areas suitable for cultivation of beans are the northern zone (Arusha, Kilimanjaro, Manyara and Tanga Regions), eastern zone (Morogoro Region), southern highlands zone, western zone (Kigoma Region) and the northwestern regions of Kagera and Mara around Lake Victoria. Although mostly a subsistence crop in many areas of Tanzania, some regions such as Kilimanjaro and Arusha commercially produce the crop (Hillocks et al., 2006).

**Figure 1:**
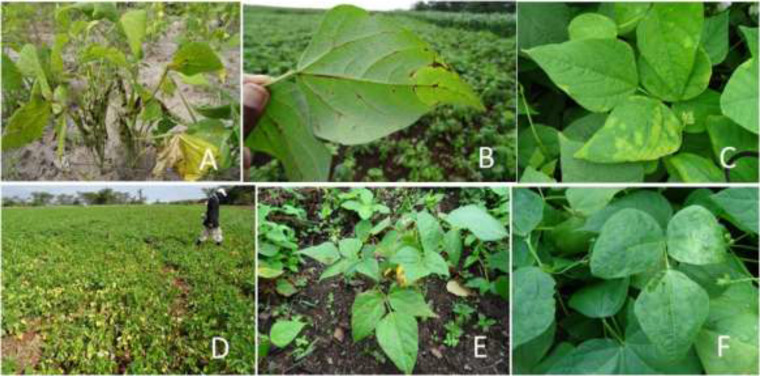
Symptoms of selected diseases of common bean in Tanzania. Anthracnose symptoms on common bean pods (**A**) and leaves (**B**) in southern highlands zone in Nkasi District; halo blight in northern zone in Karatu District (**C**); root rot in northern zone in Siha District (**D**); angular leaf spot in northern zone in Lushoto District (**E**); and virus-like mosaic symptoms in north western Kagera (**F**). All photos were taken in a country-wide common bean virus disease survey conducted during May to November, 2015.

The estimated mean dry weight yield of common bean for Tanzania is 982.5 kg/ha (FAOSTAT, 2014), which is lower than the potential yield of >1500 kg/ha (Nchimbi-Msolla, 2013). Such low yields are attributed to both abiotic and biotic factors, namely drought, pests and diseases (Hillocks et al., 2006; Mourice and Tryphone, 2012). Some of the diseases that constrain bean production in Tanzania are angular leaf spot (caused by *Phaeoisariopsis griseola*), anthracnose (caused by *Colletotrichum lindemuthianum*), root rot (caused by *Pythium* spp. and *Fusarium* spp.) and common bacterial blight (caused by *Xanthomonas axonopodis* pv. *phaseoli*). Other important diseases of common bean in Tanzania are bean common mosaic disease (caused by *Bean common mosaic virus*, BCMV; and *Bean common mosaic necrosis virus*, BCMNV), Ascochyta blight (caused by *Phoma* spp.), halo blight (caused by *Pseudomonas savastanoi* pv. *phaseolicola*) and leaf rust (caused by *Uromyces appendiculatus*) (Hillocks et al., 2006; Akhavan et al., 2013; Figure 1).

Studies have addressed some of these biotic constraints in Tanzania and solutions found for some (Fivawo and Msolla, 2011; Mourice and Tryphone, 2012; Langwerden, 2014; Kusolwa et al., 2016). There have also been reviews on different aspects of common bean production in the country (Hillocks et al., 2006; Tryphone et al., 2013) but none focused on common bean virus diseases, which can cause 100% yield loss (Worrall et al., 2015). Particularly, previous reviews have not focused on the common bean virus diseases concerning their areas of incidence, distribution and approaches for detecting their causal agents. Briefly, there has been lack of organized and collated information on virus diseases of common bean in the country and this was a motivation for this review. Given the economic importance of potyviruses in common bean production, special emphasis is on BCMV and BCMNV; however, for the first time gaps in knowledge of other common bean viruses are highlighted. Where specific information is lacking, we briefly point out information available from studies conducted in countries neighbouring Tanzania under the assumption that there are similarities in agro-ecologies and possibility of cross-border spread of virus diseases through vectors and anthropogenic activities. The aim of this review is to stimulate studies on viruses causing common bean diseases, including (1) occurrence of different viruses of common bean in Tanzania, (2) molecular characterization of the viruses that infect common beans, (3) diagnostic tools for common bean viruses and (4) options in management of common bean virus diseases in the country. Increased awareness on common bean virus diseases in Tanzania is expected to result into informed development of strategies for management of the same and thus increased production, which in turn has implication on nutrition and income.

## VIRUSES AND VIRUS DISEASES OF COMMON BEAN

Worldwide, both pathogenic and non-pathogenic viruses infect common bean crops. They include BCMV, BCMNV, *Bean golden mosaic virus* (BGMV; *Begomovirus*), *Cowpea mild mottle virus* (CPMMV; *Carlavirus*), *Cowpea aphid-borne mosaic virus* (CABMV; *Potyvirus*), *Phaselous vulgaris endornavirus* 1 (PvEV-1; *Endornavirus*) and *Phaseolus vulgaris endornavirus* 2 (PvEV-2; *Endornavirus*), *Southern bean mosaic virus* (SBMV; *Sobemovirus*), *Cucumber mosaic virus* (CMV; *Cucumovirus*), *Bean yellow mosaic virus* (BYMV; *Potyvirus*) and *Squash yellow mild mottle virus* (SYMMoV; *Begomovirus*). Some of these viruses have been detected in Tanzania and the isolates sequenced as reviewed subsequently.

## BCMV and BCMNV

BCMV and BCMNV are positive-sense single-stranded RNA viruses belonging to the genus *Potyvirus* in the family Potyviridae. The genomic RNA of BCMV and BCMNV translate into a single polyprotein that autocatalytically cleaves into 10 mature proteins: The first protein (P1), helper component proteinase (HC-Pro), third protein (P3), first 6-kDa protein (6K1), cytoplasmic inclusion (CI), second 6-kDa protein (6K2), genome linked viral protein (VPg), nuclear inclusion a (NIa), Nuclear inclusion b (NIb) and coat protein (CP). An additional short open reading frame known as Pretty Interesting Potyviridae ORF (*PIPO*) has been described in the P3 cistron (Chung et al., 2008). BCMV and BCMNV are transmitted in common bean seeds by several aphids in a non-persistent manner (Spence and Walkey, 1995). Aspects of taxonomy and transmission of these viruses were recently reviewed (Worrall et al., 2015) and are beyond the scope of this review.

The history of BCMV and BCMNV traces back to 1917 when all strains of these viruses were considered as pathogenically identical and in literature they were assigned different names including bean mosaic virus, bean virus 1 and phaseolus virus 1 (Worrall et al., 2015). According to Kulkarni and Muguga (1973), viruses causing bean common mosaic disease were reported in Kenya and Tanzania in 1936. There was interest in surveying for this disease in the years that followed.

There are several published reports of detection of viruses that caused common bean mosaic disease symptoms on common bean in Tanzania in the 1980s and 1990s (Vetten and Allen, 1991; Spence and Walkey, 1994, 1995; Myers et al., 2000). According to Vetten and Allen (1991), BCMNV and BCMV, which were then referred to as serotypes A and B, respectively, already existed in Tanzania and occurred in single or dual infections. Serological studies indicated that most isolates at that time reacted strongly to the antiserum raised to “BCMV” strain NL5 which led the authors to suspect that most isolates collected from Tanzania (also Burundi, Rwanda, Uganda and Zambia) belonged to serotype A or what is today known as BCMNV (Vetten and Allen, 1991). Isolates belonging to this group were associated with systemic vascular necrosis (also known as black root; Grogan and Walker, 1948) in common bean plants. A report of surveys and enzyme linked immunosorbent assay (ELISA) detection of BCMNV conducted during 1993 to 1998 in Tanzania, Uganda and Kenya showed that 15% (n = 1378 bean and wild legumes) of the samples were infected with potyviruses, with BCMNV accounting for 54% of this (Myers et al., 2000). The results of Myers et al. (2000) showed that incidences of BCMNV were low in Southern Tanzania but high in Northern Tanzania and even higher in samples collected from Uganda and Kenya, suggesting that distribution of BCMNV was related to altitude.

In a collaborative research project between the Sokoine University of Agriculture (SUA) and Washington State University, an isolate of the serotype A virus from common bean seed produced in Tanzania was characterized and shown to cause systemic vascular necrosis (black root) at a temperature range of 23 to 27°C (Silbernagel et al., 1986). This was the first published account of occurrence of the temperature insensitive necrosis-causing strain of BCMV in Tanzania. This strain has since been named TN1 (Silbernagel et al., 1986; Worrall et al., 2015). To date, serotype A isolates have been reclassified as BCMNV but serotype B isolates are BCMV (Worrall et al., 2015). BCMNV occurs at higher incidences compared with BCMV and its origin is believed to be wild plants in central and southern Africa (Spence and Walkey, 1995). The strains known to occur in Tanzania as determined using differential cultivars are NL1, NL3, NL5, NL8, TN1, TN2 and TN3 (Vetten and Allen, 1991; Spence and Walkey, 1994; Njau and Lyimo, 2000).

### Incidence of BCMV and BCMNV in seeds

From the early days of discovery, BCMV strains were shown to be transmitted through infected seeds (Morales and Castaño, 1987). The rates of transmission of BCMV and BCMNV strains depend on common bean genotypes (Morales and Castaño, 1987) and can be as high as 83% (Bos, 1971). Transmission of BCMNV, however, is not possible in common bean plants that have the dominant *I* gene because of the black root symptom, which results in plant death and thus no seeds for the next cropping season (Grogan and Walker, 1948). There are few published studies on seed transmission of BCMV and BCMNV in Tanzania. A comprehensive study was conducted about 17 years ago and aimed at determining the incidence of BCMV and BCMNV in seeds collected from farmers, public markets and Agricultural Research Institutes (ARIs) and wild legumes (Njau and Lyimo, 2000). There were 10,300 seeds collected in this study, representing 341 and 30 seed lots of common bean and wild legumes, respectively (Njau and Lyimo, 2000). The seeds were grown in an insect proof screen-house and the incidence of BCMV and BCMNV determined using ELISA. The two viruses were detected only in bean seed samples collected from northern and eastern Tanzania (Njau and Lyimo, 2000) and were not detected in wild legume seeds. The virus infections were more common in bean seeds collected from ARIs (in 20 out of 59 seed lots) and rare in bean seeds collected from farmers and public markets (in four out of 282 seed lots). The highest incidence for BCMNV was 36.6%, whereas it was only 12.4% for BCMV. The average incidences for both viruses were less than 8% (Njau and Lyimo, 2000).

### Alternative hosts of BCMV and BCMNV

Plant viruses, including potyviruses, can infect a wide range of hosts. BCMNV and BCMV, for instance, infect plants in at least six families (Bos and Gibbs, 1995; reviewed in Worrall et al., 2015). In Tanzania, surveys for alternative hosts of BCMNV were conducted within 1993 and 1998 (Myers et al., 2000). In their survey, Myers et al. (2000) found that several wild legume species were hosts of BCMNV: *Centrosema pubscens*, *Neonotonia wightii*, *Senna* spp., *Crotolaria* spp. and *Rhynchosia zernia*. Moreover, in artificial mechanical inoculations, BCMNV and BCMV infected five of the six legumes studied (Njau and Lyimo, 2000). The legumes infected were *Senna occidentalis*, *Senna obtusifolia*, *Cassia floribunda*, *Crotalaria* spp. and *Rhynchosia minima*. Njau and Lyimo (2000) showed that these viruses were systemic in four of the six infected plants. In Uganda, Sengooba et al. (1997) reported natural occurrence of BCMNV in *C. pubescens*, *Crotalaria incana*, *Lablab purpureus*, *Phaseolus lunatus*, *Senna bicapsularis*, *Senna sophera*, *Vigna vexillata* and also in an unidentified *Crotalaria sp*. Alternative hosts of BCMNV and BCMV including *Glycine max*, a natural host of BCMNV have been reported previously (Spence and Walkey, 1995; Worrall et al., 2015).

### Occurrence of other common bean virus diseases in the country

Worldwide, common bean is infected by a large number of both single- and double-stranded DNA and RNA plant viruses. In addition to BCMNV and BCMV, other important viruses infecting common bean are CPMMV (Mink and Keswani, 1987; Chang et al., 2013), CMV (Davis and Hampton, 1986; Njau et al., 2006), CABMV (Bashir et al., 2010), SBMV (Verhoeven et al., 2003), BGYMV (Karkashian et al., 2011), SYMMoV (Karkashian et al., 2011), PvEV-1 (Okada et al., 2013; Khankhum et al., 2015), PvEV-2 (Okada et al., 2013), BGMV and Calopogonium golden mosaic virus (CalGMV) (Diaz et al., 2002; Karkashian et al., 2011). Viruses that infect common bean in Tanzania and elsewhere are shown in Table 1.

**Table 1:** Some viruses known to infect common bean worldwide.

Virus	Abbreviation and taxonomy	Genome type^b^	Reference^*^
*Bean common mosaic virus^a^*	BCMV (*Potyvirus*; *Potyviridae*)	+ssRNA	Njau and Lyimo, 2000
*Bean common mosaic necrosis virus^a^*	BCMNV (*Potyvirus*; *Potyviridae*)	+ssRNA	Njau and Lyimo, 2000
*Cowpea mild mottle virus^a^*	CPMMV (*Carlavirus*; *Betaflexiviridae*)	+ssRNA	Mink and Keswani, 1987; Njau et al., 2006
*Cucumber mosaic virus^a^*	CMV (*Cucumovirus*; *Bromoviridae*)	+ssRNA	Davis and Hampton, 1986; Njau et al., 2006
*Cowpea aphid-borne mosaic virus^a^*	CABMV (*Potyvirus*; *Potyviridae*)	+ssRNA	Bashir et al., 2010; Patel and Kuwite, 1982
*Southern bean mosaic virus*	SBMV (*Sobemovirus*; unassigned to family)	+ssRNA	Verhoeven et al., 2003
*Bean golden yellow mosaic virus*	BGYMV (*Begomovirus*; *Geminiviridae*)	ssDNA	Karkashian et al., 2011
Calopogonium golden mosaic virus	*CalGMV* (*Begomovirus*; *Geminiviridae*)	ssDNA	Diaz et al., 2002; Karkashian et al., 2011
Squash yellow mild mottle virus	SYMMoV (*Begomovirus*; *Geminiviridae*)	ssDNA	Karkashian et al., 2011
*Phaseolus vulgaris endornavirus* 1	PvEV-1 (*Endornavirus*; *Endornaviridae*)	dsRNA	Okada et al., 2013; Khankhum et al., 2015
*Phaseolus vulgaris endornavirus* 2	PvEV-2 (*Endornavirus*; *Endornaviridae*)	dsRNA	Okada et al., 2013; Khankhum et al., 2015
*Bean golden mosaic virus*	BGMV (*Begomovirus*; *Geminiviridae*)	ssDNA	Kim et al., 1978

a These viruses have been detected in common bean or other crops in Tanzania.

* References shown are examples only and not exhaustive lists,

b ssRNA, dsRNA, ssDNA and dsDNA stand for single stranded ribonucleic acid, double stranded ribonucleic acid, single stranded deoxyribonucleic acid and double stranded deoxyribonucleic acid, respectively.

## MOLECULAR CHARACTERIZATION OF ISOLATES FROM TANZANIA

Developing strategies to manage common bean virus diseases requires knowledge of the transmission mechanism and molecular characteristics of the causal viruses. There is scanty information on molecular characterization of common bean viruses for Tanzanian isolates. Sometimes isolates are sequenced and sequences submitted in nucleotide databases without publication. Therefore, the nucleotide sequence databases were searched for availability of sequences of isolates collected from Tanzania. However, there was only one sequence for BCMNV and none for any other viruses infecting common bean in Tanzania (Table 2). This contrasted with availability of information on molecular characterization of common bean viruses in other bean growing locations, notably in Asia, Latin America, USA and Europe.

**Table 2:** The number of nucleotide sequences of selected viruses of common bean in GenBank in December 2016.

Virus name	Total number of sequences	Complete sequences	Tanzanian sequences
*Bean common mosaic virus*^a^	288	52	0
*Bean common mosaic necrosis virus*^a^	35	9	1
*Cowpea mild mottle virus*^a^	38	8	0
*Southern bean mosaic virus*^a^	13	5	0
*Cowpea aphid-borne mosaic virus*^a^	90	7	0
*Cucumber mosaic virus*^a^	3019	489	0
*Bean golden mosaic virus*^a^	173	168	0
*Phaseolus vulgaris endornavirus* 1^b^	5	3	0
*Phaseolus vulgaris endornavirus* 2^b^	2	2	0

a Pathogenic common bean virus.

b Non-pathogenic common bean viruses that are highly transmitted in common bean seeds and potentially spread all over the world.

## BCMV

A search for BCMV in the NCBI nucleotide database in December 2016 resulted in 347 genomic sequences. Individual examination of each of these sequences showed that only 288, some unverified, were submitted as being of BCMV. The remaining sequences were stated as being of Azuki bean mosaic virus, Blackeye mosaic virus, Peanut stripe virus and Dendrobium mosaic virus, which are regarded as strains of BCMV (Worrall et al., 2015). Of the 288 BCMV sequences, assuming no isolate was assigned duplicate accession numbers, only 52 sequences were complete genomes (about 10 kb) that were generated by either Sanger or next generation sequencing (NGS) techniques. Although there were isolates whose origins were not mentioned (Worrall et al., 2015), it could not be established that any of these partial or complete genomic sequences were of BCMV isolates from Tanzania.

## BCMNV

There were 34 BCMNV nucleotide sequences in the sequence databases. Of these, only nine sequences translated to yield complete polyproteins, each of which could be predicted to yield 10 mature proteins upon autocatalytic cleavage (Adams et al., 2005). One of these nine sequences (KX302007), however, was only nearly complete because a few nucleotides (12 nt) were missing at the 5′-end of the 5′-untranslated region. Moreover, only one complete sequence (Accession number HQ229995) of BCMNV isolates was originally collected from Tanzania (Larsen et al., 2011). Additionally, there was a coat protein and 3′-UTR partial sequence of what was referred to as strain TN1 (Accession number U37076) but the country of origin was not stated. However, this partial sequence was 100% identical to the corresponding genomic region in the TN1 strain of Tanzanian origin, suggesting that the sequences were the same isolate.

Previous phylogenetic analysis using these nine complete genomes of BCMNV showed that they were closely related (Worrall et al., 2015). Sequences of BCMV isolates are known to be very diverse (Worrall et al., 2015) and contain evolutionary signatures of frequent recombination events (Zhou et al., 2014). Their complete nucleotide sequence identities were computed using the BioEdit software (Hall, 1999). The nucleotide sequence identities between isolates of different strains of BCMNV were in the range of 92.5 to 100%. The sequence of BCMNV isolate from Tanzania (strain TN1) was 94.4 to 98.4% identical to complete nucleotide sequences of other BCMNV isolates (Table 3).

**Table 3:** Nucleotide sequence identities of the nine complete genomes of BCMNV isolates.

Accession/strain	AY282577	KX302007	U19287	HQ229995*	HQ229994	HQ229993	AY138897	AY864314	NC_004047
AY282577	**XX**	96.3	99.8	**98.4**	96.5	99.4	99.9	95.6	99.8
KX302007		XX	96.1	**96.0**	97.8	96.1	96.2	92.5	96.1
U19287			XX	**98.3**	96.3	99.2	99.8	95.4	100
HQ229995*				XX	**96.3**	**98.3**	**98.4**	**94.4**	**98.3**
HQ229994					XX	96.3	96.5	92.6	96.3
HQ229993						XX	99.4	95.4	99.2
AY138897							XX	95.6	99.8
AY864314								XX	95.4
NC_004047									XX

* Accession number of the only sequenced isolate of BCMNV from Tanzania. Authors and determined or genetically related strain for each sequence shown in the Table are indicated in parenthesis after each accession number: AY282577 (Unpublished; NL-3), KX302007 (Maina et al., 2016; NL-8), U19287 (Fang et al., 1995; strain NL-3), HQ229995 (Larsen et al., 2011; strain TN-1), HQ229994 (Larsen et al., 2011; strain NL-8), HQ229993 (Larsen et al., 2011; strain NL-5), AY138897 (Unpublished; NL-3), AY864314 (Larsen et al., 2005; NL-3) and NC_004047 (Fang et al., 1995; NL-3).

Studies using a differential symptoms approach have consistently placed BCMNV and BCMV into seven pathogenic groups: I to VII. Despite tight clustering of BCMNV isolates in phylogenetic analysis, they cause different symptoms on differential cultivars. However, there is very little information regarding molecular characteristics of BCMNV and BCMV isolates in Tanzania. Consequently, such aspects as selection pressure and recombination events which drive evolution of plant viruses, including common bean viruses (García-Arenal et al., 2001; Larsen et al., 2005; Feng et al., 2014; Zhou et al., 2014), have remained largely unstudied. The lack of such information means it has not been possible to use PCR-based methods for reliable detection of both viruses in plants in past and continuing breeding programmes. With evidence of high genetic variation and frequent recombination events in BCMV isolates (Larsen et al., 2005; Feng et al., 2014), the availability of many sequences from outside East Africa may be insufficient for development of strain-specific diagnostic primers. Therefore, in some studies on common bean virus diseases that were conducted in Tanzania, researchers in ARIs and universities have relied on ELISA assays to detect viruses (Vetten and Allen, 1991; Njau and Lyimo, 2000; Njau et al., 2006).

## CMV

CMV, a seed-borne virus (Davis and Hampton, 1986; Zitter and Murphy, 2009), is one of the most important plant viruses and is known to have a wide range of hosts including common bean (Davis and Hampton, 1986; Zitter and Murphy, 2009; Amayo et al., 2012). In Tanzania, CMV has been detected by ELISA in a common bean leaf sample collected from Arusha (Njau et al., 2006) and in *Vigna unguiculata*, *Cucumis sativus*, *Citrullus lanatus*, *Cucurbita pepo*, *Cucumis hystrix*, *Luffa aegyptiaca* (Sydänmetsä and Mbanzibwa, 2016) and *Solanum lycopersicum* (Nono-Womdim et al., 1996). CMV has also been detected in common bean and *P. lunatus* in samples from Zambia (Vetten and Allen, 1991) and Ethiopia (Spence and Walkey, 1995), respectively. There were no nucleotide sequences of CMV for isolates from Tanzania (Table 2).

## CABMV

CABMV is known to occur in East Africa (Bock, 1973; Orawu et al., 2013) and in Tanzania has been detected in cowpea (*Vigna unguiculata* (Patel and Kuwite, 1982; Patel et al., 1982) and common bean (Sengooba, 2001). There were 90 nucleotide sequences in the nucleotide sequence database. None of these nucleotide sequences were indicated as being of isolates from Tanzania.

## CPMMV

CPMMV belongs to the genus *Carlavirus* and is transmitted by whitefly in a non-persistent manner. It was detected in common bean plants in Tanzania in the 1980s (Mink and Keswani, 1987). Since it was only detected in and near plots of imported germplasm at the SUA, the researchers involved surmised that it was introduced from India through infected mung bean (*Vigna radiata*) (Mink and Keswani, 1987). There were nine complete (about 8 kb) and 30 partial nucleotide sequences of CPMMV in the database and none of these isolates was from Tanzania.

## PvEV-1 and PvEV-2

Two high-molecular mass dsRNA viruses, named PvEV-1 and PvEV-2 and belonging to the genus *Endornavirus* (family Endornaviridae), were recently discovered in common bean (Okada et al., 2013). These viruses are efficiently (nearly 100%) transmitted through seeds (Okada et al., 2013). The viruses have been characterized at molecular level with a total of seven sequence submissions (Accession numbers NC_023678, AB719398, AM284175, X16637, AB719397, KT456288 and KT456287) in the database but none come from Africa. Of the seven sequence submissions, two were partial sequences (Accession numbers AM284175 and X16637) closely related to the corresponding genomic region in the sequence of an isolate PvEV-1 (Accession number AB719397). There has been no survey for PvEV-1 and PvEV-2 in Africa despite availability of a molecular detection tool (Segundo et al., 2008). The lack of mention of these viruses in literature of common bean virus diseases in Africa may be because they are considered non-pathogenic and therefore of no economic significance. Moreover, it may also be due to the fact that no NGS technique has been used to detect viruses in common bean plants in Africa.

## SBMV

There are no reports of SBMV infections in common bean plants in Tanzania. Likewise, a NCBI search for SBMV sequences of Tanzanian origin showed no submissions (Table 3). This is consistent with previous reports from plant pathologists in the region (Allen et al., 1989). However, occurrence of this virus may be overlooked because it usually causes mild symptoms on common bean.

## BGMV

BGMV (*Begomovirus*) is a single-stranded DNA virus with two components (A and B) and is transmitted by whitefly (*Bemisia tabaci* Gennadius). It has been reported to infect and cause symptoms on common bean plants in the New World (Bonfim et al., 2007). Searching the nucleotide sequence databases under the search terms “bean golden mosaic virus”, followed by examining each sequence, revealed 173 sequences for BGMV. Of these, 168 were complete sequences of BGMV for DNA components A and B. There was no information about occurrence of this virus in Tanzania and searching the nucleotide databases did not reveal any sequenced isolate of this virus from Tanzania. The highest number of sequences for this virus was generated in a study conducted in Brazil (Sobrinho et al., 2014).

## METHODS FOR DETECTION OF COMMON BEAN VIRUSES

Reliable and cost-effective detection of plant pathogens, including viruses is a crucial step in management of plant diseases. There is a wide range of methods used to detect plant viruses worldwide; however, they can be categorized into two major types: Serological and molecular methods. The two most commonly used virus detection methods in laboratories across the world are ELISA and PCR (Boonham et al., 2014). There is also the use of electron microscopy for morphological identification and indicator plants in bioassays. According to Boonham et al. (2014), the adoption of the technique may be based on detection sensitivity, repeatability and reproducibility, detection in fields and resource poor locations, simultaneous detection of multiple pathogens and power to discover new pathogens. Plant pathology laboratories in Africa are normally resource poor, experience problems acquiring reagents and equipment, and have erratic supply of electricity, even with good funding, for geographical and institutional reasons.

## PCR

PCR is commonly used in detection of common bean viruses (Xu and Hampton, 1996; Melgarejo et al., 2007; Tavasoli et al., 2009; Petrović et al., 2010). A universal primer pair for detection of the potyviruses that infect common bean plants was recently published (Zheng et al., 2010). The PCR detection is more accurate and sensitive compared with ELISA and its sensitivity may be improved through use of immunocapture reverse transcription PCR for RNA viruses (Udayashankar et al., 2012). In detection of common bean viruses, PCR detection followed by sequencing of PCR products directly or after cloning in plasmids is mostly used to confirm viruses detected by ELISA (Petrović et al., 2010). Molecular-based methods have not been used to detect common bean viruses in Tanzanian laboratories. Nevertheless, the numbers of laboratories using molecular techniques (that is, PCR) are increasing in East Africa although lagging far behind laboratories in the developed world. Of the 16 national agricultural research centres in Tanzania, PCR machines (including for quantitative PCR) have been installed at only one institute, the Mikocheni Agricultural Research Institute. However, Tanzania-based international agricultural research centres such as the International Institute of Tropical Agriculture have state-of-the-art laboratories with equipment for molecular detection of plant pathogens. Through collaborative arrangements, researchers based at national agricultural research centres can access the facilities in these laboratories. Moreover, PCR can be performed at the University of Dar es Salaam, SUA and the Nelson Mandela African Institution of Science and Technology. Training, mainly since the late 1990s, has led to availability of agricultural researchers capable of running laboratories that employ molecular-based techniques in detecting plant pathogens. Moreover, sequencing facilities have been installed at different institutes in the country. Examples are sequencing facilities at Mbeya Zonal Referral Hospital Laboratory and at SUA in the College of Veterinary and Medical Sciences. Sending samples there for sequencing reduces both costs and time required to send samples outside the country. In-country sequencing reduces time for results delivery from about a week to three working days. This allows for prompt interventions in management of plant diseases as well as timely completion of laboratory-based experiments that aim at generating sequence data.

Several factors have hindered application of molecular techniques in detection of common bean viruses in the country. There has been inadequate funding, which may be attributed to more attention paid to diseases of root and tuber crops, cassava and sweet potato, compared with other crops in the region. Moreover, although BCMV and BCMNV are known to be among the main constraints to common bean production in the country, there are other pathogens that constrain its production even more (Hillocks et al., 2006). Another reason is, as shown in this review, that there are no published sequences from Tanzania for designing primers to be used in PCR detection of viruses that infect common bean in the country. It is also likely that the known high genetic diversity between isolates of BCMV, which is considered the most important virus of common bean, hinders development of molecular-based diagnostic primers. However, this may not be true for the genetically closely related BCMNV strains.

PCR detection has been shown to work for nucleic acids (both DNA and RNA) extracted from dry leaf samples (Aloyce et al., 2013). Use of dry leaves as a starting material for nucleic acid extraction is paramount for virus detection in developing countries; many leaf samples collected from fields in Tanzania are delivered to the laboratory after pressing in the herbarium. Unfortunately, the detection of common bean viruses has not been demonstrated from dry leaf samples in the country, but it is likely that viral RNA will be well preserved if leaves are carefully pressed. There are alternative means for collection of samples for nucleic acid extraction. For example, fast technology for analysis (FTA) cards have been used to preserve nucleic acids and used in fields to collect samples for laboratory analysis (Ndunguru et al., 2005; Owor et al., 2007). Moreover, leaf samples can be kept in silica gel or CaCl_2_ for desiccation (Vetten and Allen, 1991). Freeze drying ensures high-quality materials for RNA and DNA extraction, and for mechanical transmission of viruses to indicator plants, but is difficult to implement at the field level.

## NGS-based detection

In a large country like Tanzania, with common bean produced in nearly all places, the chances of occurrence of many known and unknown viruses is high. This makes it difficult to apply conventional PCR in detection of viruses that infect common bean because this would require that viral genomic sequences are known for all viruses and that several different pairs of primers are designed. Moreover, plant viruses have genomes, which are either single- or double-stranded or circular, or linear DNA or RNA. Thus, in the past, researchers devised methods to target viruses depending on the nature of their genomes (Haible et al., 2006; Balijja et al., 2008). To overcome the limitations associated with different methods in detection of plant viruses, NGS techniques have been developed. These methods are robust in detection of plant viruses from different families and have led to discovery of novel plant viruses (Adams et al., 2009; Kreuze et al., 2009; Boonham et al., 2014; Zheng et al., 2017).

Some studies conducted in or using plant samples from Tanzania have employed NGS to detect plant viruses but not those infecting common bean (Mbanzibwa et al., 2014; Ndunguru et al., 2015). Elsewhere, viruses infecting common bean have been detected and sequenced using NGS (Kehoe et al., 2014; Maina et al., 2016). In detection of plant viruses infecting crops other than common bean in Tanzania, either sequencing was done on viral small RNAs, naturally generated by plants as they defend against invading viruses, or on intact RNA (Mlotshwa et al., 2008; Mbanzibwa et al., 2014; Ndunguru et al., 2015). The NGS studies on Tanzanian plant RNA samples enabled detection of viruses not previously known to occur in sweet potato (Mbanzibwa et al., 2014) and the sequencing of complete genomes of *Cassava brown streak virus* (*Ipomovirus*) and *Ugandan cassava brown streak virus* (*Ipomovirus*), and hence studies on evolution of these viruses (Ndunguru et al., 2015). Following generation of information on these viruses, plant breeders have targeted specific species in their breeding programmes and have diagnostic tools to confirm viruses with which they challenge their breeding materials. Moreover, this has allowed development of diagnostic tools that have in turn helped pathologists to distribute clean planting material to farmers in the country.

Despite exciting opportunities in use of NGS to universally detect viruses in plants, there are challenges associated with its use in detection of plant viruses in developing countries. Firstly, there is nearly always a need for collaboration with scientists from advanced laboratories in developed countries. Finding collaborators interested in the same projects as scientists in the developing countries is not easy, but such collaboration is required in order to have access to supercomputing machines and also for backstopping in NGS data analysis. However, for small-data NGS analysis studies, such as assembly of plant virus genomes, it is possible to overcome the problem of supercomputer access through purchase of computers with relatively high computation capability, 8 TB hard disk drive (HDD) computers are sufficient for most virus genome assembly work. Moreover, installing a Linux virtual machine in 1 TB HDD or less capacity computers is sufficient for analysis of NGS data using some computer programs such as VirusDetect program (Zheng et al., 2017).

Although it is possible to assemble and map plant virus genomes using commercial packages (Kehoe et al., 2014), it is expensive to purchase such software as CLC genomic workbench and Geneious whose costs may be around USD6000 and 300, respectively, or even more depending on terms and conditions (Smith, 2014). There are also costs associated with updating of software (Smith, 2014).

Secondly, sequencing service are outsourced from outside the continent (e.g. at Fasteris in Switzerland). This requires the shipping of RNA samples on dry ice or manipulating of RNA, which may compromise results. Unfortunately, all low-cost international couriers operating in Tanzania do not accept packing using dry ice and specialist couriers that accept nucleic acids on dry ice charge over USD3000, an amount sufficient to cover vehicle hire costs for disease surveys covering a distance greater than 3000 km.

Thirdly, comprehensive countrywide surveys would require collection of many samples and their sequencing is likely to cost a huge amount of money (but some argue that it reduces costs as explained below) despite recent decreases in sequencing costs (Boonham et al., 2014). As an example, as of December 2016, sequencing of two samples cost about EUR1185 (with a 5% discount on sequencing and library preparation). Therefore, working with individual plant samples is not possible for countrywide surveys of plant virus disease incidence; however, pooling an equal amount of RNA extracted from up to 100 individual plants has enabled detection of viruses in many samples at lower cost. Unfortunately, pooling of RNA extracted from many individual plants complicates the exercise of assembling genomes of individual isolates. This is because it cannot be determined which isolates the sequences obtained relate to, unless PCR is separately done on cDNA or DNA of individual plants whose RNA was pooled in a single tube. Otherwise there is a likelihood of assembling the sequences in a manner that will create artificial recombinant sequences.

Among laboratories, the issue of costs associated with use of NGS is perceived differently. (1) Using NGS reduces the time required to detect viruses in many samples and thus reduces costs associated with laboratory work (e.g. salaries). (2) Only viruses detected by NGS can be specifically targeted in the steps that follow. Thus, no money is wasted on ordering primers or different antibodies to target many suspected viruses that may not be infecting samples. (3) Timely identification of a pathogen enables rapid intervention and thus may prevent spread of disease or damage to crops, which has cost implications. Therefore, the cost challenge on use of NGS may not apply in some cases and when decisions on its use are rationally made.

## ELISA method

ELISA has been used widely in detection of common bean viruses. It has been used to detect viruses in seeds (Klein et al., 1992; Arli-Sokmen et al., 2016) and leaf samples (Tremaine et al., 1985; Shahraeen et al., 2005; Peyambari et al., 2006; Petrović et al., 2010; Arli-Sokmen et al., 2016). In Tanzania, ELISA is the only method that has been used to detect common bean viruses in common bean, mung bean and wild plants (Mink and Keswani, 1987; Vetten and Allen 1991; Myers et al., 2000; Njau and Lyimo, 2000; Njau et al., 2006).

ELISA detection uses a polystyrene plate capable of binding antibodies with association of the enzyme-substrate reaction (Jeong et al., 2014). When performed using nitrocellulose and nylon membranes, it is known as a tissue blot immunoassay (Jeong et al., 2014) or dot immunobinding assay. With ELISA, it is possible to detect viruses in many leaf or seed samples (cost effective) in a relatively short period (normally six hours to two days). Costs and time of screening for viruses in leaf samples may be reduced further by using a mixture of antibodies in a single well for simultaneous detection of co-infecting viruses and also by detecting viruses in pooled plant leaf samples (Njau et al., 2006). However, ELISA may be less accurate and sensitive compared with molecular-based diagnostic techniques. It can fail to distinguish between strains of the same virus (Boonham et al., 2014) and in some instances the antibody may react with plant constituents. Moreover, ELISA is only useful when the virus that causes the disease is known and there are antibodies for the virus or its strains. Despite these shortcomings of ELISA, BCMNV and BCMV isolates have been consistently classified into two distinct serotypes using ELISA and these groups have been confirmed using molecular techniques. The method will inevitably continue to be used because of its simplicity (no requirement for highly trained personnel) and cost effectiveness.

Many laboratories in developing countries have the human capacity and facilities to apply ELISA for detection of plant viruses: refrigeration, incubators and spectrophotometers. Commonly, ELISA is done on fresh leaf samples; however, keeping leaf samples fresh may be difficult in large surveys for common bean viruses in Tanzania. Following surveys, it may be a long time before scientists return to laboratories for sample processing and ELISA detection. The commonly used approach in storage of collected leaf samples is placing them between print sheets and pressing in an herbarium. Lister et al. (1985) demonstrated that a *Barley yellow dwarf mosaic virus* (*Luteovirus*) survived different environmental conditions after being air-dried. Dry leaf samples of common bean have been used for ELISA detection of common bean viruses (Spence and Walkey, 1994, 1995).

### Indicator secondary hosts of viruses

Indicator secondary plants are used in bioassays and host range studies. A good example of use of indicator plants in detection of plant viruses is the detection of virus infections in sweet potato using *Ipomoea setosa*. Other commonly used secondary hosts include *Nicotiana* spp., *Petunia* spp. and *Datura* spp. Virus infections in common bean have also been detected using indicator plants (that is, bioassays). Petrović et al. (2010) identified BCMV, BCMNV, CMV and *Alfalfa mosaic virus* based on the reaction of *Glycine max*, *Lupinus albus*, *Datura stramonium*, *Zinnia elegans*, *Nicotiana glutinosa*, and *Nicotiana tabacum* var. *samsun*. In other studies, *Vigna unguiculata* var. *sinensis* was also included (Lee et al., 2015). In Tanzania, Njau and Lyimo (2000) determined reaction of selected leguminous plants to BCMV and BCMNV. Mechanical inoculation is the most commonly used approach in transmission of viruses between bean plants and secondary hosts.

Indicator plants must be carefully characterized for their reactions to a given virus isolate. The indicator plants should also be able to accumulate sufficient virus that detection is possible even for common bean material with mild symptoms or asymptomatic virus infections.

## COMMON BEAN VIRUS DISEASE MANAGEMENT STRATEGIES

### Use of disease-free seed

In areas with low disease pressure and with appropriate timing to avoid high populations of vectors, use of disease-free, certified seed leads to increased yields, all other things being equal. In Tanzania, the use of clean seeds is increasing in areas where there are seed companies or where farmers are being sensitized and supported by bean-related projects like Tropical Legume III (TLIII) and non-governmental organizations such as World Vision-Tanzania and Farm Africa. The International Center for Tropical Agriculture is supporting Tanzanian organizations and private seed entrepreneurs (e.g. Meru Agro-Tours and Consultants Co. Ltd in Arusha) to produce and market bean seeds with the aim of sustaining a bean seed delivery system. Because of these efforts, quality declared seeds (QDS) is used by farmers in three zones in Tanzania: The Lake and northern and southern highlands zones (Table 4). The commonly used QDS varieties are Jesca, Lyamungu 90, Lyamungu 85, Selian 97, Selian 94 and Uyole 03. A simple market survey conducted across the three zones showed that a kilogram of breeder seed costs around USD3.7 (TZS 6000 to 8000/-), whereas that of QDS costs USD1.2. This is affordable compared with the price of farm-saved seed of USD0.8. It should be noted that, in the past, involvement of inexperienced farmers in seed production led to seed degeneration and affected viability of the seed trade (Hillocks et al., 2006). Moreover, most farmers in Tanzania use farm-saved bean seeds from the previous harvest or purchase them from fellow farmers at local village markets, a consequence of the failure of the formal seed sector to meet the needs of smallholders for high-quality seed (Hillocks et al., 2006).

**Table 4:** Areas in Tanzania where farmers are growing QDS as of January 2017.

Agricultural zone	Region	District	Varieties grown as QDS
Lake	Kagera	Bukoba Rural, Missenyi, Karagwe and Muleba	Jesca, Lyamungu 90 and Njano Uyole
	Arusha	Arumeru, and Mondulu	Lyamungu 90
Northern	Manyara	Babati and Karatu	Jesca and Lyamungu 90
	Tanga	Kilindi and Lushoto	Jesca, Lyamungu 85, Lyamungu 90 and Selian 94
	Iringa	Iringa and Makete	Njano Uyole and Uyole 96
	Mbeya	Mbeya	Njano Uyole and Uyole 03
Southern Highland	Njombe	Njombe and Wanging’ombe	Calima Uyole, Njano Uyole, Resenda, Uyole 03 and Uyole 96
	Rukwa	Sumbawanga and Nkasi	Calima Uyole and Njano Uyole
	Songwe	Mbozi and Momba	Calima Uyole, Njano Uyole, and Uyole 96

### Planting of resistant materials

Several improved cultivars, which are tolerant or moderately or completely resistant to BCMV or BCMNV have been released in Tanzania and include Canadian Wonder, Uyole 94, Uyole 96, Lyamungu 85, Jesca, Selian 97, Rojo, Mshindi, Selian 05, Selian 06 and Cheupe (Tryphone et al., 2013). Kusolwa et al. (2016) also registered the red kidney bean germplasm line (AO-1012-29-3-3A) that has multiple virus and bean weevil (storage pest) resistances. This bean germplasm line has *I* and *bc-12* genes that confer complete resistance to BCMV and BCMNV. Many other breeding programmes are being implemented in Tanzania; a good example is an on-going programme that aims at developing multiple-resistance (including BCMNV and BCMV) bean varieties based on Mshindi, which is an improved variety obtained in crosses that involved the variety Kablanketi (Nchimbi-Msolla, 2013). Breeding for resistance against other viruses is rare. Although there has been no intensive screening for resistance against many different common bean viruses in Tanzania (but see Njau et al., 1994) there are landraces that remain symptomless in fields that also contain other plants with severe virus-like symptoms. This seems to indicate that local landraces might have resistance trait(s) to some viruses that exist in Tanzania. Screening for resistance against BCMNV (serotype A, as then called) showed that there were lines in the SUA germplasm collection that were resistant to isolates of BCMNV (Njau et al., 1994). BCMNV can be prevented from spreading through seeds by planting varieties with the dominant *I* gene. Plants with this gene are killed by BCMNV through the black root syndrome and so cannot contribute to the next generation (Grogan and Walker, 1948). Use of resistant materials is the most effective way to manage plant virus diseases worldwide. However, in most cases this is complicated by the ability of viruses, especially plant RNA viruses, to evolve so rapidly that they overcome resistance faster than breeders can release new varieties.

### Control of vectors

Common bean viruses are transmitted by insect vectors such as aphids (potyviruses), whiteflies (CPMMV and BGMV) and beetles (SBMV). Being an annual crop, vector transmission of viruses in common bean is possible through occurrence of plants infected by seedborne viruses and viruses harboured in alternative host plants, especially leguminous weeds. Indeed, low incidence of BCMNV in Malawi and southern parts of Tanzania was attributed to low vector population densities due to the high altitudes of these areas (Myers et al., 2000). Management of common bean vectors, when possible, would therefore prevent virus infections of plants grown from virus-free common bean seeds. However, the most devastating virus diseases of common bean in Tanzania are caused by BCMV and BCMNV, which are transmitted by aphids in a nonpersistent manner (Vetten and Allen, 1991; Hillocks et al., 2006). The aphid vectors take a very short time to transmit these viruses to plants such that application of insecticides is not effective. The same is true for the non-persistently whitefly transmitted CPMMV (Brito et al., 2012), which has been detected in common bean in Tanzania (Mink and Keswani, 1987; Vetten and Allen, 1991). Conversely, pesticide use may prevent or reduce infection by persistently transmitted viruses.

There are cultural practices that can prevent vectors from transmitting viruses to common bean plants. For instance, planting early in the season helps the plants escape the high aphid population period (Buruchara et al., 2010). Mulching can reduce potyviruses infections and it is thought that the lack of bare soil reduces aphid landings during crop emergence and before the canopy has fully formed (Kirchner et al., 2014 and references therein).

### Field sanitation and avoidance of alternative hosts

Vector-mediated transmission of viruses between alternative hosts and common bean plants has been studied, although not in Tanzania (Spence and Walkey, 1995). The inoculation of BCMV and BCMNV isolates from common bean onto other legume plants caused disease symptoms on the latter (Njau and Lyimo, 2000). This indicated that the BCMV and BCMNV isolates from common bean successfully infected certain wild legumes and could be transmitted back to common bean plants by vectors. Moreover, Myers et al. (2000) reported BCMNV natural infections in several cultivated and uncultivated legumes in Tanzania. Recently, CMV was detected in cucurbits (Sydänmetsä and Mbanzibwa, 2016). Therefore, transmission of viruses between common bean plants and alternative hosts is possible. Indeed, aphid transmission of BCMV and BCMNV from wild legumes to common bean, secondary hosts and wild legumes has been demonstrated (Spence and Walkey, 1995). It is reasonable to assume that avoidance of leguminous weeds in and around common bean fields and proper handling of crop residues would reduce the spread of diseases within and between fields. Indeed, removing weeds and non-common bean weeds has been recommended for management of bean diseases (Buruchara et al., 2010). Management of alternative hosts for common bean viruses is likely to be complicated by farmers’ preferences for mixed cropping and the medicinal value attached to some potential alternative hosts. Field sanitation is, however, easy to achieve in areas where farmers produce QDS for marketing because QDS production involves, field isolation by distance and regular inspections by seed industry regulators, the Tanzania Official Seed Certification Institute.

## CONCLUSION

This review aimed at assembling the information on virus diseases of common bean and the molecular characterization and detection of the responsible viruses in common bean in Tanzania. Also reviewed was the progress on management of common bean virus diseases. The writing of this review was mainly motivated by the fact that most studies on common bean virus diseases are inaccessible, fragmented and some are of unknown date; however, the information contained therein would be helpful in management of common bean virus diseases. It is acknowledge in the study that some literature might have been missed during writing this review but the information presented can guide in development of integrated disease management strategies. It is anticipated that this review will revitalize interest in studying common bean virus diseases beyond the common mosaic diseases (that is, BCMV and BCMNV) by identifying the gaps in research on common bean virus diseases, the neglected but economically important viruses such as CPMMV, CMV and SBMV)

It is evident from the information obtained from literature and stakeholder consultation concerning viruses and virus diseases of common bean in Tanzania that there is scanty information concerning distribution, occurrence, characterization and detection for nearly all viruses known to cause diseases of common beans in many other countries. Although viruses that infect common beans in other countries have been characterized at the molecular level and diagnostic tools developed, in Tanzania such information is rare and when available, for example for BCMNV (Silbernagel et al., 1986), is too old (30 years) and involves very few isolates. A sequence of common bean virus collected from Tanzania may have been missed because the origins of isolates were not supplied for all nucleotide sequence submissions; however, this would not appreciably change the results from the few sequenced isolates from Tanzania. Molecular characterization of viruses depends on availability of funds, which is affected by national priorities, political drivers as well as private donors’ funding interests.

There was also scant information on alternative hosts, incidence and distribution of common bean viruses in the country. Such information is required to develop integrated pest management strategies. For instance, such information would guide deployment of specified resistant planting materials for different agro-ecological zones because information on occurrence of viruses and their strains would determine the appropriate cultivar to distribute to farmers in each area.

In addition, availability of information on genetic diversity would equip common bean breeders with the tool for decisions in their breeding programmes. Whereas several common bean viruses are known to cause indistinguishable symptoms on common bean plants, different genotypes of common beans may respond differently to viruses or strains of the same virus (Feng et al., 2014). Literature searching revealed a lack of knowledge on molecular characteristics of the common bean virus isolates in Tanzania; there was only one sequence and that was for BCMNV. Consequently, current identification of virus isolates for evaluating resistance of bean genotypes is based on response of differential cultivars. There is an inherent danger in this because viruses can cause unexpected phenotypes (Feng et al., 2014).

Since molecular characterization has not been done for viruses that infect common bean in Tanzania, it was recommended that NGS be considered and used in any future surveys for these viruses. This will enable detection of both known and unknown, DNA and RNA viruses. This can be followed by the use of ELISA and development of specific and degenerate primers for detection of specific or group of viruses using the sequences generated using NGS techniques.
